# The alkaline treatment and its influence on the physicomechanical properties of plantain pseudostem fibers - A comparative study of treated and untreated fibers

**DOI:** 10.1016/j.heliyon.2025.e41843

**Published:** 2025-01-09

**Authors:** Oswaldo Hurtado-Figueroa, Humberto Varum, María Isabel Prieto, Romel J. Gallardo Amaya, Alfonso Cobo Escamilla

**Affiliations:** a Escuela Técnica Superior de Edificación, Universidad Politécnica de Madrid—UPM, 28040 Madrid, Spain; b CONSTRUCT-LESE, Departamento de Engenharia Civil, Faculdade de Engenharia da Universidade Do Porto, FEUP, 4200-465 Porto, Portugal; c Departamento de Tecnología de La Edificación, Universidad Politécnica de Madrid—UPM, 28040 Madrid, Spain; d Grupo de Investigación en Construcción, Geotecnia y Medio Ambiente—GIGMA, Universidad Francisco de Paula Santander Ocaña—UFPSO, 546552 Ocaña, Colombia

**Keywords:** Plantain pseudostem, Alkaline treatment, NaOH, Mechanical properties, Surface modification, Vegetable fibers

## Abstract

Fibers were extracted from the pseudostem of the plantain of the variety Hartón, belonging to the order Zingiberales, family Musaceae, genus Musa, species Musa paradisiaca. The fibers were exposed to alkaline treatment to improve their physicomechanical properties for use as reinforcement in green composites. NaOH solutions of 4 %, 5 %, and 6 % by weight were prepared. The fibers were immersed for 2 h in each solution. Fibers were washed with acetic acid to remove NaOH residues and dried at room temperature. Weight loss and lignin, cellulose, and hemicellulose content were evaluated. FTIR, tensile, and elongation tests were performed. SEM images and EDS mapping allowed to conclude the phenomena that occurred in the treatments. The untreated fibers obtained a tensile strength of 37.23 MPa, in contrast to 104.26 MPa obtained by the fibers treated with 5 % NaOH solution. It was concluded that the partial elimination of impurities and waxes improved the mechanical behavior of the fibers by 180 %. Regarding elongation, the fibers treated with 4 % NaOH solution obtained the best result with 3.7 %. It was determined that the partial elimination of lignin favors elongation by 48 %. Excessive hemicellulose removal was found to cause fibril separation and detachment. Considerable contraction of vascular bundles occurred. Roughness in the fibers surface topography were improved.

## Highlights

• Lignin and hemicellulose act as coupling agents between fibrils
• Controlled alkaline treatment improves the mechanical properties of vegetable fibers
• Lignin content positively influences the % elongation of vegetable fibers
• The rupture of support fibers in vascular bundles accelerates their contraction
• High concentrations of NaOH cause detachment of fibrils

## Graphical abstract

**Figure:**
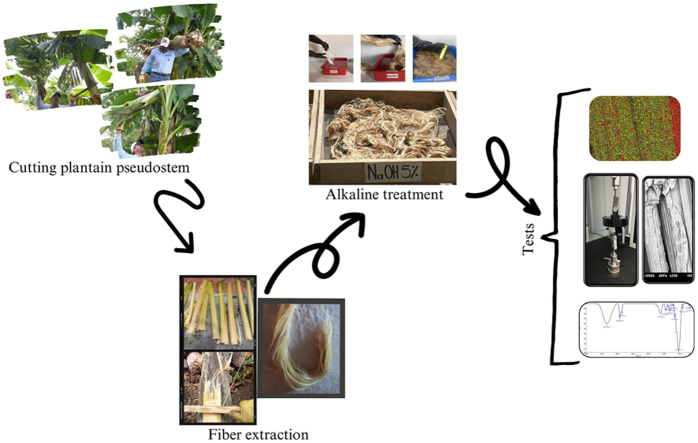

## 1 Introduction

The worrying climate situation caused by the irrational use of non-renewable natural resources, the emission of greenhouse gases, and the industrialized manufacture of consumer products is causing concern in different countries regarding the control and mitigation of environmental impact. Satisfying the needs generated by the uncontrollable increase in the world's population currently entails the use of bad environmental practices commonly carried out by omission or simply by ignorance [1,2].

For this reason, the reduction of polluting waste and the correct final disposal of waste materials from the different production processes is the main objective of the industrial sector in search of environmentally friendly alternatives [3].

Consequently, promoting the ecological philosophy in clean production frames the path of respectful engineering leading to specialized research efforts that aim to apply corrective measures. This research is focused on increasing the environmental performance objectives oriented to the study and analysis of renewable natural elements for their implementation in composite material [4,5].

In this way, and to make significant contributions to the environmental issue, scientists propose research objectives for the development of composite materials of ecological origin or green composites whose particularity is represented by its biodegradable matrix and the use of vegetable fibers as reinforcement elements [6].

It should be noted that the environmental characteristic of the green composites denotes other particularities in its matrix and reinforcement. Particularities of which the economy, renewable extraction, abundance in its availability, and minimum or null energetic expenses in its obtaining and preparation, mark differential guidelines at the moment of its election as starting elements for its conformation [7].

Regarding the matrix, polymeric materials are the most commonly used in the development of Green composites due to the ease of processing at low temperatures, compared to ceramic and metallic matrix [8].

However, the manufacture of green composites involves the use of a naturally occurring polymeric matrix with degradable or biodegradable characteristics due to the particular dependence on petroleum for the manufacture of conventional polymeric materials [9].

Therefore, a biodegradable polymeric matrix is understood as one whose accelerated degradation is carried out by a mixed microbial population in a humid, warm, and aerobic environment under controlled conditions. Factors that fragment the polymer, converting it into food and energy sources for microorganisms [10].

However, research is developing matrices of natural origin with organic materials. Such is the case of the study carried out by the authors of this article, who improved the mechanical properties of clayey matrix mixtures by incorporating cassava starch and carboxymethylcellulose [11].

As for the vegetable fibers reinforcement, its organic particularity makes it the best choice for the conformation of ecologically friendly materials because it does not need to be recycled or reused due to its renewable and compostable characteristics [12].

### 1.1 Vegetable fibers as reinforcing elements

Technological advances aimed at satisfying human needs have made progress in several areas, the most significant of which are health, telecommunications, and materials. Technological advances have led to improvements in medical treatments, facilitated interpersonal communication, and improved physicochemical and morphological properties of materials allowing a wide range of applications [13].

As a result, technological advances in the industrial sector have developed materials with high serviceability features that improve their performance and durability. Such is the case of composite materials reinforced with carbon fibers, kevlar, petroleum-based polymeric fibers, and glass fibers, among others, which in combination with metallic, ceramic, or polymeric matrices make the latest generation of composite materials [14].

However, even though these technologies generate composite materials with special characteristics, they present problems in their recyclability and degradation due to the inorganic origin of their components and the complexity of their separation. Characteristics that add to the environmental impact provided by the industrial sector [15].

For this reason, research has concluded that vegetable fibers are the best option to replace the inorganic fibers traditionally used to manufacture composite materials. Thus, the plant-based and renewable nature of vegetable fibers provides several environmental benefits [16]. The benefits of their biodegradability, physical-mechanical properties, abundance, low cost, and rapid growth are part of the arguments that promote vegetable fibers as reinforcement in composite materials [17]. This combination of mechanical and environmental properties motivates research that studies vegetable fibers from a sustainable point of view, adding value to the byproducts generated by the agroindustrial sector [18].

In context, vegetable fibers are classified according to their origin or part of the plant from which they are extracted. Thus, the vegetable fibers are classified into 2 groups. Group 1, wood fibers such as those present in hardwoods and softwoods. Group 2, non-timber fibers, to which vegetable fibers extracted from seed or fruit, stem bark, leaves, stems, and of herbaceous nature belong [19].

Similarly, the biological origin of vegetable fibers represents a special feature in their agricultural or forestry origin, making it an unlimited renewable resource that can provide raw materials for the production of substitute materials for those traditionally used [20].

These environmental characteristics have led researchers to identify the scope for different plant fibers as replacements for synthetic fibers by encouraging the effective cultivation of plants, trees, and fruits and using agricultural residues to provide employment possibilities in underdeveloped regions [21].

Jute, sisal, pineapple, flax, hemp, and plantain vegetable fibers have the potential for implementation as substitutes for synthetic fibers due to their unique properties. That is, their low density, biodegradability, non-corrosive nature, abundant availability and ease of processing favor their use in the production of environmentally friendly composite materials [22].

However, one of the main problems with plant fiber-reinforced composites is to ensure the continuity of their physical and mechanical properties. This is because the properties of vegetable fibers vary with their cultivation process [23].

The plantain is a favorite fruit in tropical areas. However, the banana plant only bears fruit once in its life; it is cut after harvest, and only 40 % of it is properly utilized, leaving the remaining 60 % as waste rich in cellulose, hemicellulose, and natural fibers. The pseudostem represents 75 % of the unusable plant and 25 % of the useable bunch [24].

Plantain pseudostem fibers present interesting characteristics for their use as reinforcement elements in materials for non-structural applications. Their low density and considerable mechanical strength allow their consideration as reinforcement in composite materials with environmentally friendly ecological characteristics. This type of application in the elaboration of novel materials allows the use of the great % of agro-industrial waste generated in the post-harvest of the fruit.

Ehi et al. [25] developed polymer matrix composites reinforced with banana pseudostem fibers to evaluate the impact strength, toughness, and acoustic properties of the material. The authors concluded that the addition of 20 % by weight of banana fibers considerably improved the mechanical and acoustic properties of the composite material due to the energy absorption of the experimental mixtures. The research identified the importance of the fiber-matrix interaction in the increase of the mechanical properties of the experimental mixtures. The adhesion phenomenon is caused by the roughness of the fiber surface.

Al-Daasa et al. [26] improved the physico-mechanical characteristics of banana pseudostem fibers by chemical processing. The authors concluded that the elimination of the impurities present in the fibers improved the surface structure favoring the anchorage with the matrix increasing its mechanical resistance. The mechanical anchorage between the matrix and the fiber was generated by the adherence of the matrix in the rough areas caused by the alkaline treatment after the removal of wax, lignin, and hemicellulose.

Lakshumu et al. [27] elaborated a laminar composite with groundnut shell ash and plantain fibers in a 15 % by-weight polymeric matrix. The authors concluded that the mechanical properties of the composite material increased due to the energy dissipation of the composite by the action of the plantain fibers. This characteristic favors the use of these fibers in the manufacture of interior and decorative components of vehicles.

Boopalan et al. [28] tested the mechanical and thermal properties of hybrid composites of polymeric matrix reinforced with banana and jute fibers. The authors concluded that the 50/50 ratio in addition of banana and jute fibers concerning the polymeric matrix favored the thermal and mechanical properties of the composite. The mechanical properties of the experimental mixtures increased due to the intertwining of the cellulose microfibrils because of the controlled loss of lignin.

**Fig. 1:**
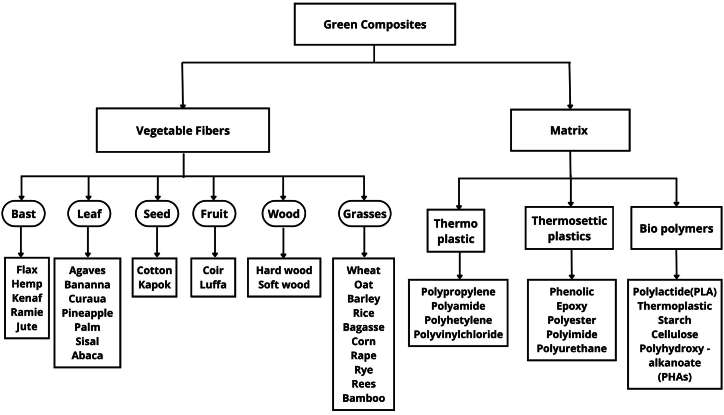
Types of vegetable fibers and matrixes used in green composites [30].

Igea et al. [29] studied the physicomechanical and thermal properties of earth bricks reinforced with banana pseudostem fibers. The authors concluded that the reinforced earth bricks increased their tensile strength by 53 % and their compressive strength by 33 %. Similarly, it was shown that the reinforced bricks increased their ultimate strength by 18 % to the unreinforced bricks. The increases in mechanical properties of the earth bricks were caused by the structural reinforcement caused by the interfacial adhesion of the plant fibers with the clay matrix. Fig. 1 describes the types of plant fibers and polymeric matrices most commonly used in the manufacture of environmentally friendly composites. The figure shows the species and plant parts from which the fibers can be extracted. Likewise, the polymeric matrices commonly used in the manufacture of green composites are shown [30].

However, regardless of the origin of the vegetable fibers to be used as reinforcement, it must guarantee performance according to the desired service performance for the green composites [31]. Behavior that is directly related to the content of its 3 fundamental polymers, lignin, cellulose, and hemicellulose, the condition of its surface topography, and its resistance to external stresses [32].

For this reason, vegetable fibers are subjected to controlled physical or chemical treatments that guarantee the improvement of their physicochemical properties. Treatments that alter its components to increase their adhesion and mechanical resistance to guarantee the performance of the green composites under external stresses [33].

### 1.2 Interaction vegetable fiber-matrix

The difference in mechanical behavior between the fiber and the matrix that make up a composite material from an individual perspective, fixes the research interest of specialists who seek synergy between components to provide better resistance values to the action of external stresses [34].

In other words, the matrix enables composite material processability and serves as the basis for the guidance and protection of the fibers. However, by itself, it does not present significant values in its mechanical resistance. In contrast, the reinforcing fibers are responsible for supporting the mechanical stresses to which the composite material is subjected [35].

That is to say, since the matrix presents a different mechanical behavior than the one supported by the reinforcing fibers, it is necessary to guarantee the transmission of stresses between the fiber (Fr) and the matrix (Mx), ensuring the service performance of the composite material [36]. This mechanical action is commonly known as fiber-matrix interaction (FMI).

**Fig. 2:**
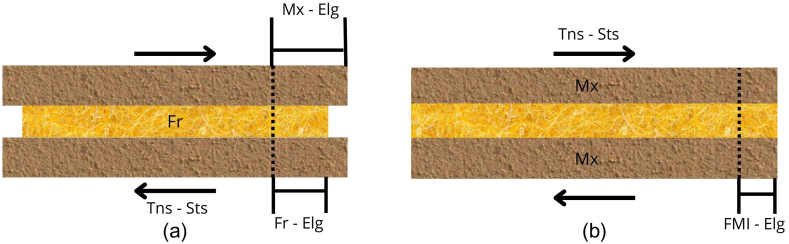
Stress effect in composite materials with and without fiber-matrix interaction [37]. (a) individual elongation matrix and fiber. (b) Joint deformation of matrix and fiber.

Fig. 2 shows the FMI in a composite material subjected to tensile stress (Tns-Sts). The behavior of composite material with and without FMI is shown. Fig. 2a shows the individual elongation of the matrix (Mx-Elg) and the individual elongation of fiber (Fr-Elg) when composite material has no FMI. On the other hand, Fig. 2b shows the behavior of composite material with FMI where the joint deformation of Fr and Mx (FMI-Elg) is presented.

To enable the isodeformation condition in a composite material through the synergy of the elements causing a uniform deformation between the FMI in response to external forces, 2 processes can be performed to significantly increase the adhesion between the Fr and Mx surfaces. I) Mechanical interaction process (Mcn), which consists of improving the surface topography of the Fr by physical or chemical treatment, allowing mechanical anchoring between the components. II) Chemical interaction process, which allows chemical anchoring between Fr and Mx through weak or strong chemical bonding (Cbg), depending on the origin of the components [38].

**Fig. 3:**
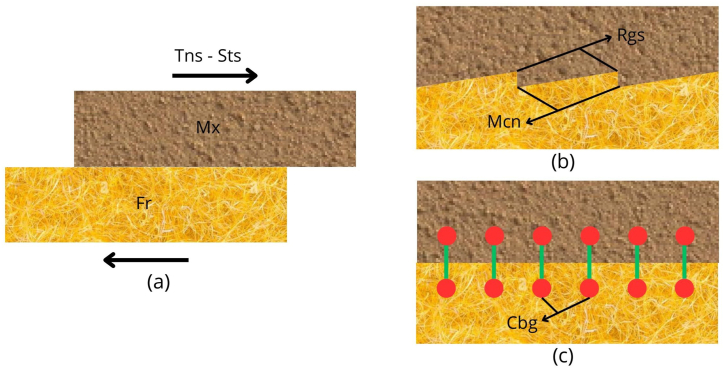
Treatment effects on fiber-matrix interaction. (a) Individual displacement. (b) Mechanical interaction process. (c) Chemical bonding.

Fig. 3 shows the influence of the processes in the FMI. Fig. 3a shows the individual displacement of the Mx and Fr of the composite material before Tns-Sts due to the lack of FMI. Fig. 3b shows the Mcn due to roughness (Rgs) resulting from physical or chemical treatment. Fig. 3c shows the FMI by Cbg resulting from chemical treatment.

The present study shows the results obtained from the mercerization treatment applied to plantain pseudostem fibers of the Hartón variety. In the study, the fibers were submerged for 2 h in NaOH concentrations of 4 %, 5 %, and 6 %. The alkaline treatment was applied to the fibers to improve their physicomechanical properties to consider their use as reinforcement fibers in green composites.

The study evaluated the loss of mass of the chemically treated fibers. The lignin, cellulose, and hemicellulose content was determined. The tensile strength and elongation of the fibers were tested. The influence of the chemical treatment was evaluated by Fourier transform infrared spectroscopy.

The observations of the phenomena that occurred were carried out by Scanning Electron Microscope. With the observation of the longitudinal and transversal images, the effect of the alkaline treatment on the surface topography of the fibers was determined. This characteristic is of interest when evaluating the adhesion phenomena between the matrix and the vegetable fiber.

The elemental chemical composition of the treated fibers was identified by energy-dispersive X-ray spectroscopy. This observation facilitated the interpretation of the phenomena through the incidence of the change of the elemental chemistry present on the surface of the treated fibers on the physical and mechanical characteristics of the fibers.

The study evaluated the alkaline treatment conditions by identifying the characteristics that can cause a reverse effect on the cellulose structure leading to a decrease in the mechanical properties of plant fibers. In other words, the influence of chemical treatments on the removal of lignin, and hemicellulose has direct effects on the decrease of physicomechanical properties of vegetable fibers.

Just plantain pseudostem fibers of the Hartón variety were evaluated in the research. This is one of the different varieties of the Musaceae family. Thus, the results of the influence of the mercerization process on the fibers of the Hartón variety are published, showing the phenomena that occurred during the chemical process to record data for future research on the use of pseudostem fibers of this variety in the manufacture of green composites.

## 2 Chemical treatments

The final performance of the composite material from its design is directly related to the physicochemical properties of its reinforcing [39]. The origin and nature of the reinforcing Fr grants particular characteristics that must be analyzed to guarantee the best performance and anchorage with the Mx [40].

That is, the incompatibility between Mx and Fr substantially affects the mechanical properties of composite material [41]. It is for this reason, that for the use of vegetable fibers as reinforcing elements, their chemical composition must be considered, of which the content of lignin, cellulose, and hemicellulose generate values of differential interest [36]. These chemical characteristics play an important role in influencing the mechanical behavior of vegetable fibers. Consequently, these 3 key polymers have an impact on increasing the absorption of moisture from the environment by increasing the volume of the vegetable fibers. A characteristic that significantly reduces its ability to withstand stresses due to the weak FMI due to the hydrophilic nature of the vegetable fibers [42].

Table 1 shows the percentage by weight (wt/%) of lignin, cellulose, and hemicellulose of vegetable fibers commonly used as reinforcement in green composites manufactured in different investigations. However, the primary chemical composition of vegetable fibers may vary due to their natural origin. In this sense, external factors such as the cultivation and harvesting process, type of fertilization, soil type, climate, and even the irrigation system can alter the physicochemical properties of the vegetable fibers [39].

**Table 1:** Cellulose, lignin, and hemicellulose content in vegetable fibers [45].

Vegetable fibers	wt/%		
	Cellulose	Lignin	Hemicellulose
Bagasse	55.2	25.3	16.8
Bamboo	26–43	21–31	30
Flax	71	2.2	18.6–20.6
Kenaf	72	9	20.3
Jute	61–71	12–13	14–20
Hemp	68	10	15
Ramie	68.6–76.2	0.6–0.7	13–16
Abaca	56–63	7–9	20–25
Sisal	65	9.9	12
Coir	32–43	40–45	0.15–0.25
Oil Palm	65	29	–
Pineapple	81	12.7	–
Curaua	73.6	7.5	9.9
Wheat straw	38–45	12–20	15–31
Rice husk	35–45	20	19–25
Rice straw	41–57	8–19	33

For this reason, the information consulted in previous research should only be reviewed as a reference in terms of the processes carried out. Assuming the similarity of data as an initial basis for further research should be avoided [43].

For this reason, and regardless of the origin of the vegetable fibers to be implemented, it must be analyzed to obtain current data regarding its chemical composition and mechanical behavior [44].

Table 1 presents the chemical composition of vegetable fibers commonly used in green composites. Fr type and wt/% of cellulose, lignin, and hemicellulose content are displayed [45]. Data that can be used as a starting point for further research.

Due to the need to modify the characteristics of vegetable fibers to improve FMI by controlled reduction of its fundamental polymer content and improvement of its surface roughness, there are physicochemical methods that can be implemented [46].

However, due to the cost and energy expenditure required for the development of physical methods in which the Fr is intervened with machinery or specialized processes such as thermomechanical to saturate the Fr and open its microfibrils [47], or mechanical processes with which it can reduce its size, reduce its diameter, or simply improve its surface topography by increasing its Rgs to ensure adhesion with the Mx, chemical treatments are presented as the best option when choosing the right method [48].

Table 2 describes in general terms the particularities of the treatments on vegetable fibers. The table shows the main characteristics of each treatment and recommendations for use according to the expected results.

**Table 2:** Overview of chemical treatments for vegetable fibers.

Treatment	Description
Organosilane	The implementation of organosilane improves the mechanical behavior in vegetable fibers due to the coating of pores forming lattice networks in its surface topography. However, organosilane involves a special process of which the use of $C_{2}H_{6}O$ , agitation, pH range and treatment temperature are differential factors that significantly influence the achievement of the expected results [[49], [50], [51], [52]].
Acetylation	Acetylation treatment can be enhanced by incorporating other chemicals into your solution. In turn, increasing the solution temperature can add value to the process. However, the controlled environment plays a very important role to avoid deterioration of the Fr by considerably reducing its mechanical performance [[53], [54], [55], [56]].
Alkaline	Alkaline treatment is presented as the process commonly implemented in the improvement of vegetable fibers intended to be used as reinforcement in green composites. Likewise, this alkaline treatment is widely used as a pretreatment accompanying subsequent chemical treatments focused on increasing the mechanical properties of vegetable fibers. However, care must be taken in controlling the % NaOH in the solution due to the degradation that vegetable fibers can present because of the degree of concentration and exposure time of in the solution [[57], [58], [59]].
Benzoyl	The use of benzoyl presents a preconditioning of the vegetable fibers with NaOH prior to use. Particular cases in which the benzoyl solution is accompanied by NaOH are also reported. Controlling the concentrations of chemical elements in the solutions has a direct impact on the physicomechanical results of the vegetable fibers [[60], [61], [62]].
Potassium Permanganate	It may be noted that the use of $KMnO_{4}$ requires pretreatment of the vegetable fibers with NaOH. However, the use of the $KMnO_{4}$ solution in acetone is implemented without prior treatment of the vegetable fibers. The potassium permanganate is characterized by the low concentration of $KMnO_{4}$ in the solution, a particularity that marks the control of the % $KMnO_{4}$ in the treatment in order to avoid the deterioration of the vegetable fibers [[63], [64], [65]].
Stearic Acid	The use of stearic acid in vegetable fibers presents different application methods that can be combined with an alkaline pretreatment which improves its surface topography by increasing the mechanical behavior. However, the values of increase in the physicomechanical characteristics of the vegetable fibers treated in previous studies should be considered to deciding to implement stearic acid in future research [[66], [67], [68], [69]].
Polymer Coating	Polymer coating is presented as an excellent treatment option to improve the properties of vegetable fibers to be implemented as reinforcing in green composites manufacture. However, polymer coating turns out to be an elaborate method that, in addition to the need for pretreatment, requires the use of a series of chemicals to achieve the final objective. For this reason, these particularities of treatment require the availability of resources, which can be significant depending on the amount of vegetable fibers that needs to be treated [[70], [71], [72]].
Esterification	Esterification treatment comprises a process of special attention involving a series of chemical elements as a pretreatment of the vegetable fibers improving its properties for the implementation of other chemical treatments. This feature, frames the use of additional resources for their enlistment. However, the evaluation of physicomechanical changes in vegetable fibers treated in previous research allows the choice of esterification for implementation in future research [[73], [74], [75]].
Fungal	Due to its biological nature, the use of fungal treatment becomes a process with minimal environmental impact. However, the implementation of biological material involves elaborated and specialized processes on which the cultivation of the strain depends. These processes can add costs at the time of treatment. In turn, the control of the exposure time of the vegetable fibers before the treatment directly affects the expected mechanical result due to the progressive enzymatic action of the Fungal treatment on the vegetable fibers [[76], [77], [78], [79]].
Bleaching	The bleaching treatment is a simple process that can be implemented in previously treated Fr or just washed directly with $H_{2}O$. In addition to improving the visual appearance of the vegetable fibers, the treatment generates considerable increases in mechanical performance. However, in the case of pretreated vegetable fibers, their mechanical characteristics depend on the concentration, temperature, and duration of pretreatment [[80], [81], [82]].

The treatments described above frame particular processes that can make a difference when choosing the appropriate treatment to be implemented in future research. However, even though they are similar in their effect on surface topography and mechanical resistance in vegetable fibers, the availability of resources and specialized laboratories facilitate the choice of treatment to achieve the objectives set out in the research development. For this reason, the analysis of the individual characteristics of each process is crucial to evaluating its implementation [83].

## 3 Materials and methods

### 3.1 Materials

The plantain plant used in the research was the variety Hartón, belonging to the order Zingiberales, family Musaceae, genus Musa, species Musa paradisiaca [84]. The pseudostem plantain was recovered from the crops present in the municipality of Zulia in the departamento Norte de Santander, Colombia. Same location where the authors of this article took samples for the rice straw mercerization process [85].

The plantain pseudostem was cut after bunch harvesting. The estimated time for harvesting the plantain bunch of the Hartón species is 365 days according to specifications and crop conditions.

For the chemical treatment, 99 % NaOH was used for the alkaline solution and 98 % $CH_{3}COOH$ for the washing solution. Alkaline treatment was performed in 3 plastic trays with a capacity of 20 L (*l*). The solution was prepared in H2O. The pH of the solutions was monitored by means of a digital pH meter model Ph-002.

### 3.2 Methods

#### 3.2.1 Plantain pseudostem fibers

Plantain pseudostem fibers (PPF) were extracted by hand. After extraction, the PPF were washed with $H_{2}O$ to remove impurities. After washing, the PPF were dried for 3 days at room temperature.

#### 3.2.2 Alkaline treatment

For alkaline treatment, 3 wt/% NaOH solutions were prepared at different concentrations. NaOH4%, NaOH5% y NaOH6%. The solution was prepared in 75 % of the container capacity (15l) in order to avoid overflow at the time of immersion of the PPF.

For the calculation of the solution quantities, the value of 998 g/l corresponding to the density of H2O at 20 °C was taken because the temperature of H2O at the time of preparing the solutions was 21 °C ± 1 °C.

The calculation of the wt/% of NaOH in solutions was performed with Eq. (1). Equation used in the research conducted by authors Hurtado-Figueroa et al. [85].

(1) $$\%\;Mass=\frac{solute\;mass}{Solution\;mass}x100$$

Replacing the data in Eq. (1) yielded the calculation of wt/% NaOH in the solutions.

With Eq. (2) the 4 wt/% NaOH in the alkaline solution was determined.

Solute: 625 g (NaOH)

Solvent: 15l ∗ 998 g = 14970 g (H2O)

Solution: 14970 g + 625 g = 15595 g

(2) $$\%\;Mass=\frac{625}{15595}x100$$

The same equation was used to determine the corresponding calculation for the wt/% of the 5 % and 6 % concentrations with the wt of 790 g and 955 g, respectively.

For each solution, 600 g of PPF were prepared. 4 % by wt of untreated PPF (PPF-Ut) was used to determine its moisture content prior to immersion in alkaline treatment. Each solution was immersed in 576 g ± 2 g of PPF. The exposure time of the PPF in each of the solutions was 2 h. After the exposure time of the PPF in the alkaline treatment, they were washed in $CH_{3}COOH$ solution at 0.2 % by volume to eliminate NaOH residues. Washing was completed until a neutral pH was identified in the wastewater. After washing with $CH_{3}COOH$ solution, the treated PPF (PPF-t) were dried for 3 days at room temperature.

NaOH concentrations and exposure time of PPF in alkaline treatment were referenced from research conducted by authors Narayana and Rao [86] and Cadena et al. [87]. Research where the authors concluded that NaOH concentrations above 8 % significantly impair the mechanical behavior of the Fr.

Similarly, research conducted by the authors Mbouyap et al. [88] concluded that exposure times longer than 5 h decrease the physicomechanical properties of vegetable fibers. In contrast, other studies indicated that NaOH solutions between 4 % and 6 % and exposure times of 1 h, 2 h, and 3 h greatly improve the physicomechanical characteristics of vegetable fibers [[89], [90], [91], [92]].

**Fig. 4:**
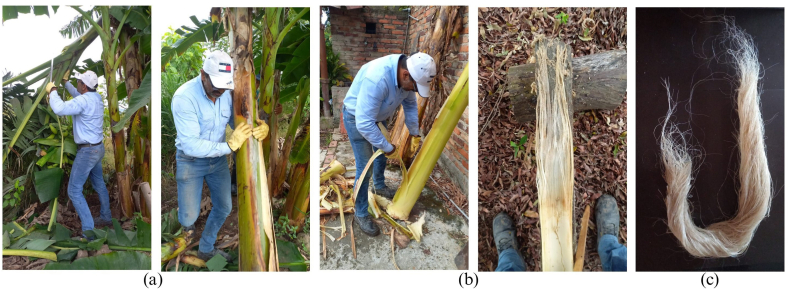
Plantain pseudostem fibers extraction process. (a) Cutting of plantain bunch and pseudostem. (b) Manual fibers extraction. (c) Extracted fibers.

Fig. 4 shows the PPF extraction process. Fig. 4a shows the cut of the bunch and the pseudostem of the plantain plant. Fig. 4b shows the manual extraction of PPF. Fig. 4c shows the extracted PPF.

**Fig. 5:**
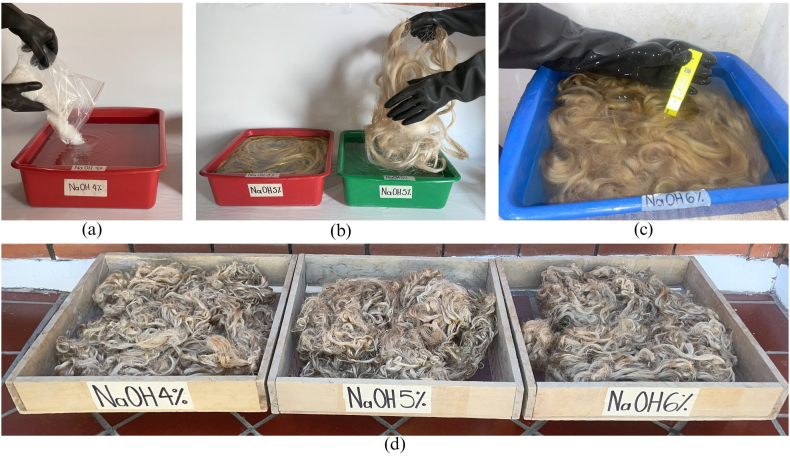
Alkaline treatment of plantain pseudostem fibers. (a) NaOH solution. (b) Immersed fibers. (c) Washing process. (d) Drying process.

Fig. 5 shows the alkaline treatment performed on the PPF. Fig. 5a shows the preparation of the NaOH. Fig. 5b shows the immersion of PPF in NaOH solutions. Fig. 5c shows the PPF-t washing process with H2O in $CH_{3}COOH$ solution. Fig. 5d shows the drying process of PPF-t at room temperature.

Table 3 describes the NaOH solutions. The table presents the solutions, the exposure time and the identification of the PPF-t.

**Table 3:** NaOH % and exposure time plantain pseudostem fibers.

Solutions	Exposure time	Identification
NaOH4%	2 h	PPF-t/ NaOH4%
NaOH5%		PPF-t/ NaOH5%
NaOH6%		PPF-t/ NaOH6%

#### 3.2.3 Weight loss

Before starting the alkaline treatment, the PPF-Ut were checked for moisture content and then weighed and immersed in the respective solution. Similarly, the PPF-t were tested for moisture content after the drying process. With similar data on the moisture contents of PPF-Ut and PPF-t, the weight of PPF-t was determined. This process was carried out to correctly identify the weight loss of the PPF after each of the alkaline treatments.

#### 3.2.4 Lignin, cellulose and hemicellulose

To identify the influence of alkaline treatment on the lignin, cellulose, and hemicellulose content in PPF, the neutral detergent fiber (NDF) [93], acid detergent fiber (ADF) UNE EN ISO 13906:2009 [94] and cellulose content tests were performed. Tests carried out by the Animal Nutrition Laboratory of the Universidad Francisco de Paula Santander- Cúcuta-Colombia.

#### 3.2.5 Fourier transform infrared spectroscopy

Due to the organic nature of the PPF, a fourier transform infrared spectroscopy (FTIR) test was performed to identify the impact of alkaline treatment on the chemical composition of the PPF-t. The test was performed with the Spectrum 100, PerkinElmer. Working range (450 cm-1 to 4000 cm-1). The FTIR was performed by the Characterization Laboratory of the Composite Materials Group (LaC-GMC) of the Universidad del Valle - Colombia.

#### 3.2.6 Tensile and elongation

The mechanical strength of PPF-Ut and PPF-t was tested using the tensile and elongation test (Tns & Elg). The test was performed based on the Colombian Technical Standard (NTC) 386:2011 [95] and ASTM D-2256/D2256M − 21 (2022) [96]. The equipment used in the test was 250 N Tension - Universal 376 Testing Machine Lloyd Instruments EZ20- CRE. 10 samples of each of the PPF-Ut and PPF-t were tested to average their results. The test was performed by the materials quality laboratory of the Textile and Industrial Management Center of the Servicio Nacional de Aprendizaje (SENA) – Medellín.

#### 3.2.7 Scanning Electron Microscope & energy-dispersive X-ray spectroscopy

To determine the effect of alkaline treatment on the longitudinal and transverse surface of PPF-t, scanning electron microscopy (SEM) was performed. Observations against which the structural difference of PPF-Ut and PPF-t can be compared. The test was carried out with Jeol JSM 6010LA equipment. The preconditioning and conditioning of PPF-Ut and PPF-t were performed according to ASTM D1776/D1776M − 20 [97].

The same equipment was used to perform the energy dispersive x-ray spectroscopy (EDS) test. Mapping that identified the elemental chemistry of the analyzed PPF-Ut and PPF-t surfaces. EDS mapping was able to determine the effects of alkaline treatment on the samples analyzed. The tests were carried out by the technological services laboratory of the Textile and Leather Manufacturing Center – SENA-Bogota.

**Fig. 6:**
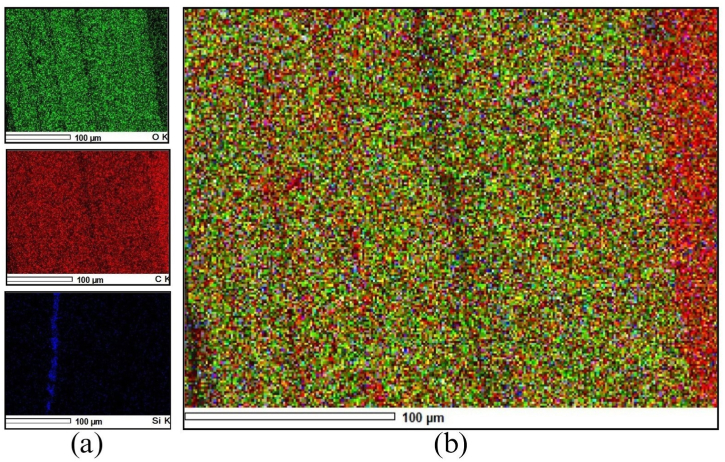
Energy-dispersive x-ray spectroscopy test. (a) Chemical element colors. (b) Mapping.

Fig. 6 shows the data obtained from the EDS test of the PPF. Fig. 6a shows the color identification of elemental chemical elements. Silica (Si) is identified by the color blue, Carbon (C) by the color red, and Oxygen (O) by the color green. Fig. 6b shows the EDS mapping.

**Fig. 7:**
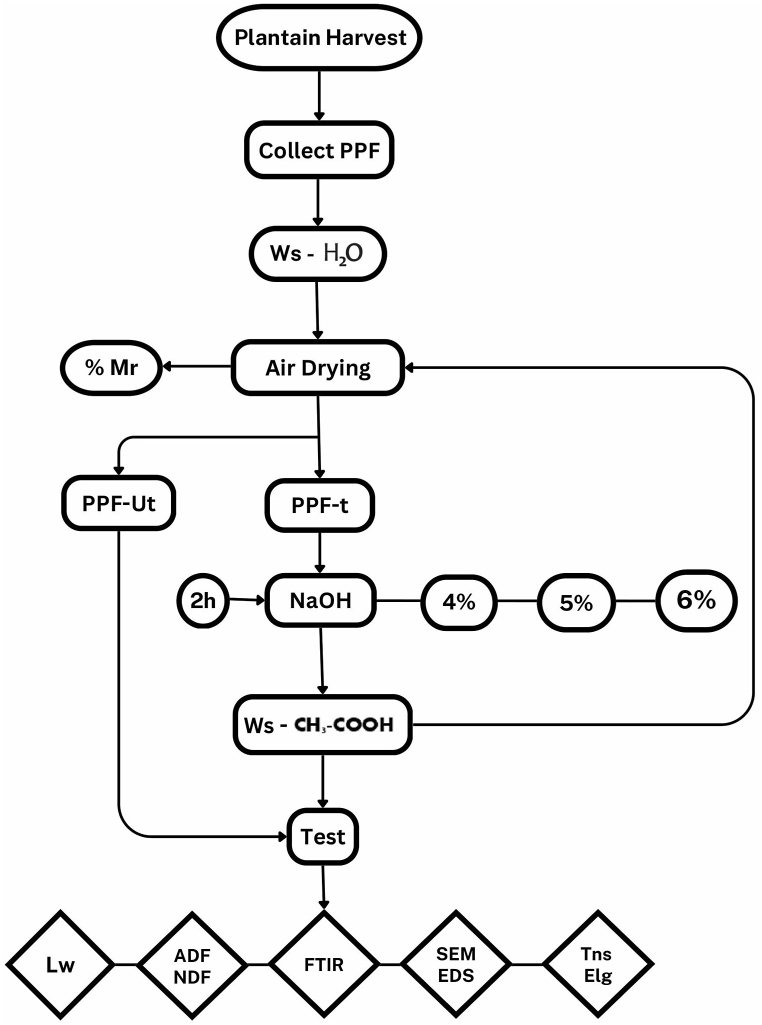
Graphical representation of the research process.

Fig. 7 summarizes graphically the processes carried out in the research. The process begins with the extraction of the PPF and ends with the performance of the tests and the interpretation of the phenomena that occurred.

## 4 Results and discussion

### 4.1 Weight loss test

The moisture content test before and after alkaline treatment allowed to correctly identify the weight loss of the PPF-t. Table 4 shows the weight loss presented by PPF-t. The number of washes (Ws) is shown. Weight in grams. Moisture percentage (%Mr). Percentage of weight lost (%Lw). Results with which you can easily calculate the amount of material to be treated to obtain the desired mass of material.

**Table 4:** Weight loss of treated plantain pseudostem fibers.

PPF-t	Incoming			Outgoing			%Lw
	Washes	%Moisture	Weight	Washes	% Moisture	Weight	
PPF-t/ NaOH4%	2	7.55	577.4	2	7.71	480.2	16.83
PPF-t/ NaOH5%	2	7.60	578.2	2	7.18	464.8	19.61
PPF-t/ NaOH6%	2	7.58	576.8	2	7.20	458.7	20.47

The %Lw corresponds to the removal of lignin, hemicellulose, wax and impurities present in the plantain pseudostem fibers. The table shows the influence of the concentration of the alkaline treatment on %Lw. In this sense, the PPF-t/ NaOH4% sample presented the lowest result with a difference of 3.64 %Lw concerning the PPF-t/NaOH6% sample. The difference between the PPF-t/ NaOH5% and PPF-t/ NaOH6% samples was 0.86 %Lw. This characteristic allows for identifying the optimum concentration of NaOH necessary to improve the surface topography of plantain pseudostem fibers, avoiding damage due to exposure of fibers to high alkaline concentrations.

### 4.2 Lignin, cellulose and hemicellulose content

The change in the molecular structure of PPF caused by alkaline treatment allowed the breaking of the lignin and hemicellulose bonds and their partial removal leaving the cellulose Fr exposed. Quantification of these 3 key polymers in PPF-Ut and PPF-t was performed by NDF and ADF tests.

Eq. (3) was used to calculate the % lignin using the ADF test as a starting point. Eq. (4) was used to calculate the % hemicellulose by subtracting the ADF from the NDF. Eq. (5) was used to determine the % cellulose by subtracting the lignin and hemicellulose from the NDF. To check the result obtained in Eq. (5), Eq. (6) was used, where the ADF was subtracted from the lignin.

(3) (ADF)−(Cellulose)=Lignin

(4) (NDF)−(ADF)=Hemicellulose

(5) (NDF)−(Lignin)−(Hemicellulose)=Cellulose

(6) (ADF)−(Lignin)=Cellulose

Table 5 lists the % cellulose, lignin, and hemicellulose calculated from the NDF and ADF tests. With the values presented in the table, the phenomena presented in the Tns & Elg test can be qualitatively interpreted. The table shows the % increase in cellulose due to the elimination of lignin and hemicellulose caused by the alkaline treatment.

**Table 5:** Cellulose, lignin, and hemicellulose content.

PPF	NDF	ADF	%		
			Cellulose	Lignin	Hemicellulose
PPF-Ut	85.60	74.10	64.30	9.80	11.50
PPF-t/ NaOH4%	89.45	80.30	72.20	8.10	9.15
PPF-t/ NaOH5%	91.45	84	76.40	7.60	7.45
PPF-t/ NaOH6%	88.45	83.35	79.15	4.20	5.10

Similarly, the % of lignin in PPF-t can be interpreted. This property increases the stretching capacity of the vegetable fibers. In the book, Physical and Mechanical Properties of Natural Fibers, published by author [20], the author describes that the % concentration of lignin increases the % elongation of vegetable fibers. A characteristic that favors the mechanical behavior of the vegetable fibers, giving ductility to the green composite.

**Fig. 8:**
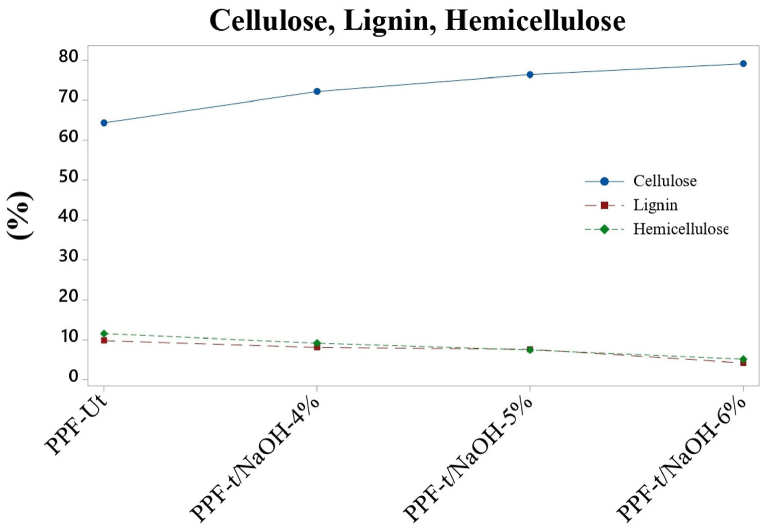
Lignin, cellulose, and hemicellulose content.

Fig. 8 shows graphically the cellulose, lignin, and hemicellulose content of the PPF-Ut and each of the PPF-t. The figure shows that the increase in the % of cellulose is inversely proportional to the % of lignin and hemicellulose. This characteristic is due to the elimination of these 2 key polymers in the vegetable fibers because of alkaline treatment.

### 4.3 Fourier transform infrared spectroscopy test

The FTIR test showed characteristic features of lignocellulosic material due to the vibrational bands of the main functional groups and chemical components [98]. Similarly, the variation of lignin, cellulose, and hemicellulose in each of the treatments to which the PPF was exposed could be identified.

**Fig. 9:**
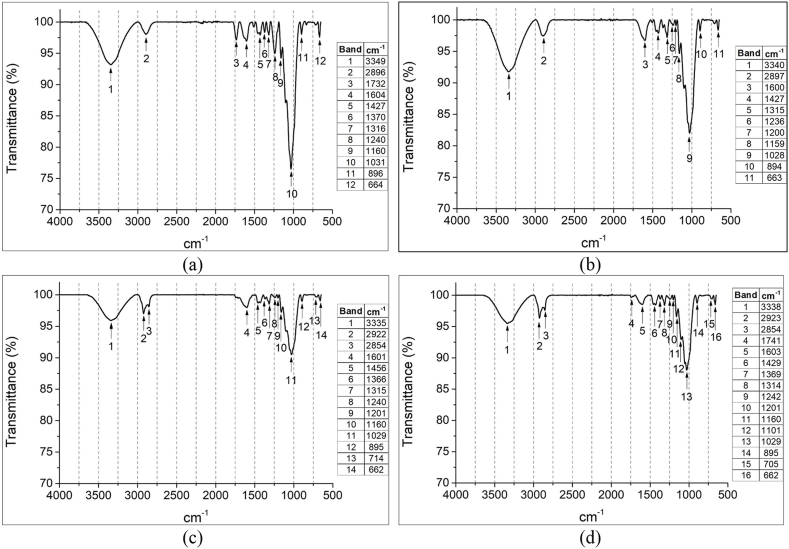
Fourier transform infrared spectroscopy test. (a) PPF-Ut. (b) PPF-t/ NaOH4%. (c) PPF-t/ NaOH5%; (d) PPF-t/ NaOH6%.

Fig. 9 shows the spectra obtained from the FTIR tests. Fig. 9a shows the spectrum of the PPF-Ut. Fig. 9b shows the PPF-t/ NaOH4% spectrum. Fig. 9c shows the spectrum of the PPF-t/ NaOH5%. Fig. 9d shows the spectrum of PPF-t/ NaOH6%.

In the 4 samples evaluated it was possible to identify the shift of the bands 3335 cm-1 to 3350 cm-1 due to the presence of some hydroxyl groups of cellulose and the aromatic stretching vibration of the lignin [99].

The spectra also show a high stretching activity of OH groups in these bands, a phenomenon that occurs after the alteration of impurities with cellulose (Fig. 9b–c, and Fig. 9d), a conclusion that was reported in the study conducted by Zhou et al. [100].

The broadband in the region corresponding to 3400 cm-1 is characteristic of the stretching vibrations of the hydroxyl groups. The bands present in the region between 3335 cm-1 and 2924 cm-1 correspond to the cellulose [87].

For this reason, these bands are more visible in the spectra of PPF-t/ NaOH5% (Fig. 9c) and PPF-t/NaOH6% (Fig. 9d) due to the removal of lignin and impurities, a phenomenon that exposes the cellulose microfibers that compose the PPF.

The spectrum shows the broadening of the absorption bands of the aromatic rings in the region corresponding to 1425 cm-1 and 1605, a phenomenon caused by the removal of the lignin caused by the alkaline treatment.

Shrinkage of the bands 1236 cm-1 to 1250 cm-1 in the treated samples is shown (Fig. 9b–c, and Fig. 9d). This feature evidencing the elimination of the hemicellulose. A similar phenomenon was reported by the authors Parre et al. [101] who indicated that the 1250 cm-1 band corresponds to the presence of hemicellulose.

The spectra generated in the region between 1500 cm-1 and 800 cm-1 generally identify lignocellulosic materials due to the vibrations associated with the C-H, C-O, C-O-C, C-C-O groups characteristic of lignin, cellulose, and hemicellulose [88].

There was a reduction in transmittance in the bands present in the 1700 cm-1 and 1400 cm-1 regions where the lignin, and hemicellulose functional groups are present due to the elimination of impurities present in the surface topography of the PPF [100].

Alkaline treatment can increase the crystallinity of the cellulose component of vegetable fibers by improving its physicomechanical characteristics [102]. However, the type of alkaline solution and the exposure time of the Fr in the alkaline treatment become important variables when choosing the ideal treatment to achieve the desired mechanical behavior of the vegetable fibers.

### 4.4 Tensile & elongation test

The tensile test performed on PPF-t/ NaOH4% obtained a 48.67 % increase in resistance compared to PPF-Ut. This value identifies PPF-t/ NaOH4% as the PPF-t with the lowest result obtained. In contrast, PPF-t/ NaOH5% and PPF-t/ NaOH6% obtained increases in tensile strength of 180 % and 167.25 %, respectively. Results indicating PPF-t/ NaOH5% as the PPF-t with the highest result obtained.

The phenomenon of increased tensile strength of PPF-t compared to PPF-Ut was reported by the research done by authors Ernest and Peter [61] who applied chemical modification agents to PPF. The authors concluded that the removal of lignin, hemicellulose, and wax from the outer surface of Fr exposes cellulose microfibrils allowing their mechanical entanglement by increasing the number of interaction sites. This physical characteristic considerably improves the mechanical properties of vegetable fibers by promoting the transmission of stresses with the matrix.

In terms of elongation, PPF-t/ NaOH4% obtained the highest result with an increase of 48 % compared to PPF-Ut. PPF-t/ NaOH5%, and PPF-t/ NaOH6% obtained increases of 40 % and 12 %, respectively. These values indicate that PPF-t/ NaOH5%, is the sample with the closest % to that obtained by PPF-t/ NaOH4%. This decrease in % elongation in PPF-t was reported in the research conducted by authors Al-daas et al. [35] who evaluated the effect of alkaline treatment on PPF used for polymer matrix composites. The authors indicated that alkaline treatment reduces the presence of lignin, hemicellulose, wax, and impurities on the surface of PPF-t by changing the properties of PPF. This phenomenon increases the % of cellulose exposing the short length crystallines leading to an increase in their rigidity. This characteristics explaining the decrease in % elongation in PPF-t as an effect of vegetable fibers exposure in alkaline treatment with higher NaOH concentrations [103].

The alkaline treatment promotes the formation of three-dimensional networks due to the bonding of different polymeric chains caused by the dissolution of the lignin present in the surface topography of PPF. A feature that substantially increases the strength of the PPF-t. However, prolonged exposure of PPF in alkaline treatment with high concentrations of NaOH aggressively disintegrates the components present on the surface, generating a progressive deterioration of the cellulose microfibrils, a phenomenon that causes a decrease in the mechanical properties of the PPF-t. Similar conclusions were reported by Valášek et al. [59] in their research on Abacá and Coir Fr. The authors concluded that Abaca Fr required less exposure time in alkaline treatment to improve their mechanical properties in contrast to Coir Fr.

Table 6 shows the results obtained in the Tns & Elg test. The average (Av) Force at break and % elongation are shown. The % Coefficient of Variation (%Cv) is displayed to analyze the deviation of the data. Maximum (Max) and minimum (Min) values are shown.

**Table 6:** Force at break & elongation results.

PPF	Force at break (N)				Elongation (%)			
	Av	%Cv	Max	Min	Av	%Cv	Max	Min
PPF-Ut	1.4	54.47	2.6	0.6	2.5	42.21	4.8	1.3
PPF-t/ NaOH4%	1.5	38.70	2.3	0.9	3.7	40.63	6.7	1.9
PPF-t/ NaOH5%	2.2	40.06	3.0	0.9	3.5	29.41	5.5	1.9
PPF-t/ NaOH6%	2.0	45.08	3.6	0.8	2.8	22.98	3.8	1.9

Table 7 shows the data used to determine the tensile strength of the vegetable fibers. The table lists the average diameter. The test length is presented. The average area is shown. The force at break (FaB). The tensile strength (TeS). The calculation of the TeS of the PPF was performed using Eq. (7).

(7) $$TeS=\frac{FaB}{Area}$$

**Table 7:** Tensile strength results.

PPF	Av				
	Diameter (mm)	Length (mm)	Area (mm^2^)	Force at break (N)	Tensile Strength (MPa)
PPF-Ut	0.219	80	0.0376	1.4	37.23
PPF-t/ NaOH4%	0.186		0.0271	1.5	55.35
PPF-t/ NaOH5%	0.164		0.0211	2.2	104.26
PPF-t/ NaOH6%	0.160		0.0201	2	99.50

**Fig. 10:**
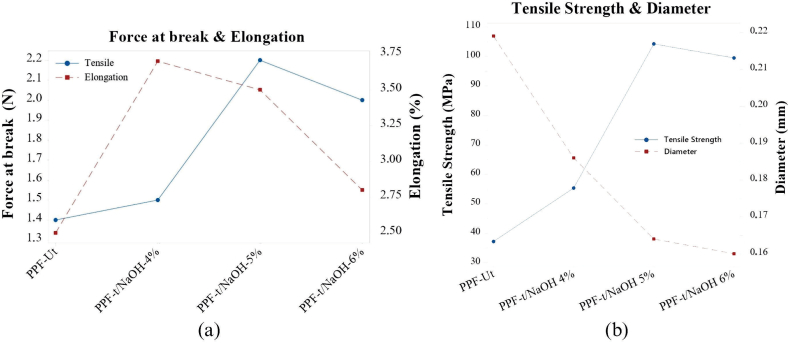
Comparative figures. (a) Force at break vs elongation. (b) Tensile strength vs diameter.

Fig. 10 shows graphically the results generated in the Tns & Elg test. Fig. 10a shows the low FaB obtained by PPF-Ut due to the natural content of lignin, cellulose, and hemicellulose, the 3 fundamental polymers present in vegetable fibers.

The spacing of the plotted points representing the results obtained for PPF-t/NaOH4% and PPF-t/NaOH6% is shown. This mechanical difference which is attributed to the effect of the amount of NaOH and exposure time of PPF on alkaline treatment.

The figure presents the closeness of the plotted points representing the results obtained by PPF-t/NaOH5%. This proximity is related to the chemical modification of the PPF components due to the removal of lignin, hemicellulose, and wax from their surface favoring the interlacing of the cellulose microfibers and increasing their mechanical properties.

Fig. 10b presents the TeS results compared to the PPF diameter. The figure initially shows a scissor effect represented by the position of the indicators. The inversely proportional relationship between the percentage reduction in fiber diameter and the increase in TeS is shown. Effect generated by NaOH concentration in the alkaline solution. It shows the low TeS of PPF-Ut being the PPF with the largest diameter. In contrast, the 15.06 % reduction of the PPF-t/NaOH4% diameter increased the TeS by 48.67 %. In the same vein, the PPF-t/NaOH5% showed a reduction in diameter of 25.11 % while increasing its TeS by 180 %. However, the PPF-t/NaOH6% had the smallest diameter compared to the other PPF showing a reduction of its TeS of 4.56 % compared to the diameter obtained by the PPF-t/NaOH5%.

These variables that were presented in the comparative analysis between diameter and TeS are attributed to the loss of lignin and hemicellulose. The pattern of loss is due to its elimination because of the concentration of the alkaline solution. Although the removal of these 2 polymers improves the physicomechanical properties of the vegetable fibers, their loss in excess impairs it mechanical behavior due to the exposure of cellulose fibrils favoring their separation and subsequent weakening.

Table 8 presents a summary of the data obtained from the physicochemical and mechanical tests performed on the PPF. The table shows the % of cellulose, lignin, and hemicellulose content of PPF-Ut and PPF-t. The % of Lw is showing. The Av of the results obtained in the Tns and Elg test. The FaB. The TeS.

**Table 8:** Physicomechanical and chemical PPF results summary and its comparison with other vegetable fibers.

PPF	%				Av			Reference
	Cellulose	Lignin	Hemicellulose	Weight loss	FaB (N)	Elg (%)	TeS (MPa)	
PPF-Ut	64.30	9.80	11.50	–	1.4	2.5	37.23	Present work
PPF-t/ NaOH4%	72.20	8.10	9.15	16.83	1.5	3.7	55.35	
PPF-t/ NaOH5%	76.40	7.60	7.45	19.61	2.2	3.5	104.26	
PPF-t/ NaOH6%	79.15	4.20	5.10	20.47	2.0	2.8	99.50	
Coir fiber-Ut	39.18	38.1	15.88	–	–	16.03	71.41	[33]
Coir fiber-t/ NaOH5%	42.75	23.55	8.71	–	–	17.03	55.63	[33]
Hemp-Ut	68	10	15	–	–	1.6	690	[45]
Hemp-t/ NaOH22%	–	–	–	–	–	2.34	828	[45]
Pineapple crown leaves-Ut	18.93	18.80	13.53	–	–	–	–	[46]
Pineapple crown leaves-t/ NaOH10%	57	0.67	17.50	–	–	–	–	[46]
PPF-t/ NaOH2.5%	–	–	–	18.8	1.8	–	–	[54]
Coir fiber-t/ NaOH5%	–	–	–	–	0.95	–	0.52	[59]
Abaca fiber-t/ NaOH5%	–	–	–	–	0.019	0.005	0.0015	[59]
Sugar palm fiber-t/ NaOH18%	–	–	–	48.96	–	–	–	[62]
Coir fiber-t/ NaOH15%	22.2	37.5	12.7	–	1.57	–	52.65	[63]
Rice Straw-t/ NaOH1%	44.26	11.54	18.7	25.96	–	3.8	37.3	[85]
Rice Straw-t/ NaOH2%	45.35	9.85	17.55	28.39	–	5.1	59.7	[85]
Rice Straw-t/ NaOH3%	45.6	8.75	16.85	34.90	–	5.2	27.4	[85]
Urena lobata fibers-Ut	56.42	23.05	13.61	–	–	–	–	[89]
Urena lobata fiber-t/ NaOH6%	59.85	26.16	15.47	–	–	–	–	[89]
Red banana peduncle-Ut	72.90	15.99	–	–	–	1.57	440	[91]
Red banana peduncle fiber-t/ NaOH5%	79.13	12.3	–	–	–	2.90	650	[91]

The table compares the results obtained in the present work with other vegetable fibers chemically treated with NaOH at different % for 2 h. The comparative results coincide in the inversely proportional relationship between the reduction of the % of lignin and hemicellulose and the increase of NaOH concentration. Similarly, the directly proportional relationship between the increase in cellulose % and the increase in NaOH concentration is presented. This phenomenon is caused by removing lignin and hemicellulose by the alkaline treatment resulting in the exposure of the microfibrils that make up the cellulose fibers. Regarding Elg % and TeS, the results varied due to the type of fiber studied. The variety of vegetable fibers studied and chemically treated with NaOH allows the comparative analysis of the properties evaluated in each sample to determine the physicochemical alteration generated by the alkaline treatment.

### 4.5 Scanning Electron Microscopies & energy dispersive X-ray spectroscopy observations

The surface topography of PPF-Ut and PPF-t were analyzed by SEM and EDS tests to determine the influence of alkaline treatment on the PPF surface.

**Fig. 11:**
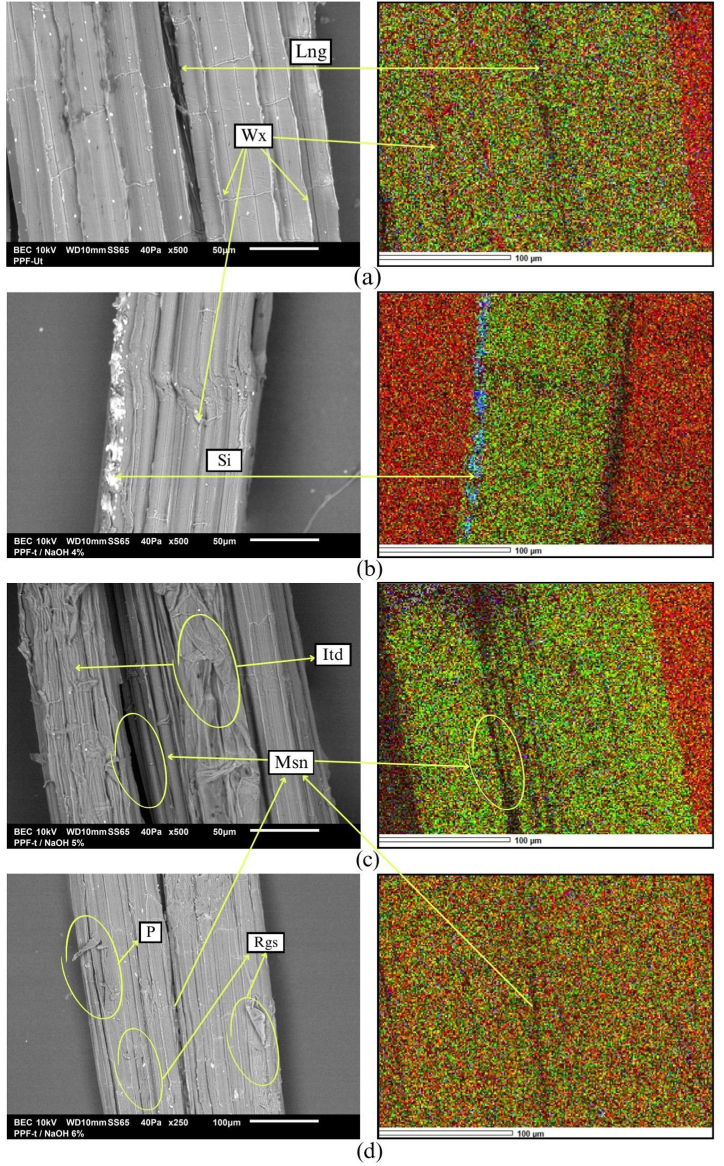
Longitudinal observations SEM-EDS. (a) PPF-Ut. (b) PPF-t/ NaOH4%. (c) PPF-t/ NaOH5%. (d) PPF-t/ NaOH6%

Fig. 11a shows the SEM observation of the PPF-Ut. The bonding action between Fr that causes the lignin. Wax (Wx) and impurities in the surface topography that coat and protect the cellulose Fr are shown. Characteristics that prevent interaction between Fr, which is reflected in its low mechanical resistance to external stresses. A similar feature was reported by the authors Gholampour and Ozbakkaloglu [104] who in their review article indicated the consequences of the presence of foreign bodies in the surface topography of vegetable fibers on the low results obtained before mechanical strength tests.

Fig. 11b shows the PPF-t/ NaOH4%*.* It shows mineralized bodies in Si layers due to the cleaning action of the alkaline treatment [85]. A reduction in the Wx content is observed at the beginning of the exposure process of the cellulose Fr. However, the little effect of alkaline treatment on the structure and composition of the PPF allowed obtaining the highest % Elg compared to the other PPF due to the lignin content still present in the sample (see Table 6). This phenomenon was reported by the authors Al daas et al. [35] who identified the importance of lignin content in the % Elg of PPF-t with alkaline treatment.

Fig. 11c shows the PPF-t/ NaOH5%*.* A remarkably clean surface is observed due to the removal of Wx and impurities. The exposure of cellulose macrofibers is observed. The separation of fibrils (Msn) is evident due to the removal of lignin and hemicellulose. It shows the mechanical Interlaced (Itd) of fibrils caused by their exposure as a result of the removal of the lignin. A characteristic that favored the increase of Tns & Elg (Fig. 10). This phenomenon coincides with the results reported by Ezeamaku et al. [105] who in their study of PPF concluded that the controlled removal of lignin, hemicellulose, and Wx allows the interaction between fibrils causing considerable increases in the mechanical strength of PPF-t.

Fig. 11d shows the PPF-t/ NaOH6%.The presence of pores (P) is evidenced due to the rupture of Fr of cellulose. A phenomenon caused by the high concentration of alkaline treatment. Msn and Rgs areas caused by the removal of lignin, hemicellulose, and Wx are observed. EDS mapping shows the absence of Si and other elements due to the notorious dispersion of O and C, elements that constitute the cellulose. This impact caused by the excessive exposure of PPF on chemical treatments was concluded in the research conducted by authors Temitayo et al. [106] who indicated the influence of chemical treatments and exposure time of vegetable fibers in the considerable reduction of their physicomechanical properties.

Alkaline treatment partially eliminates the lignin present on the surface and at the junction between PPF fibrils. Phenomenon that decreases the adhesion between fibrils generating their separation. A characteristic that causes an Rgs surface favoring FMI interaction. In not-so-aggressive alkaline treatment, the separation of fibrils allows Itd to increase its % Elg.

Lignin and hemicellulose are responsible for the cohesion and compaction of the microfibers present in the cell wall of plants, thus achieving their structural capacity under compressive stress. However, the presence of these 2 essential polymers in vegetable fibers prevents FMI when implemented as reinforcement in green composites conformation [107]. For this reason, its disposal must be controlled by avoiding aggressive chemical treatments that may cause irreparable damage to cellulose components.

**Fig. 12:**
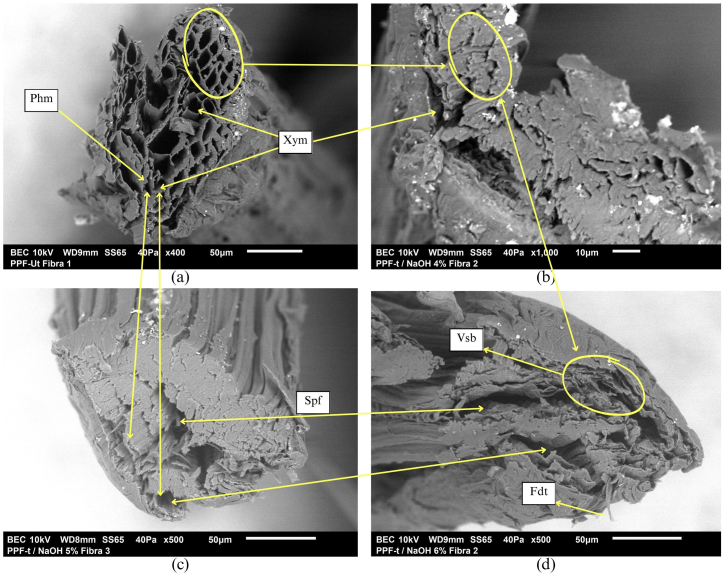
Cross-sectional observation SEM. (a) PPF-Ut. (b) PPF-t/ NaOH4%. (c) PPF-t/ NaOH5%. (d) PPF-t/ NaOH6%

Fig. 12 presents the SEM image corresponding to the cross-section of the PPF. Fig. 12a shows the image corresponding to the PPF-Ut where the conductive tissue can be easily identified. Xylem (Xym) and phloem (Phm) are observed. Components of great importance in the transport of nutrients from the root to the other parts of the plant. In addition to their function as conductive tissue, Xym and Phm are part of the plant's support mechanism that counteracts stress caused by external agents. This observation is in agreement with research by Mbouyap et al. [88] who observed tube-shaped fibril structures by SEM test.

Fig. 12b shows the cross-sectional image of the PPF-t/ NaOH4%. The image clearly shows the action of contraction and deformation of the vascular bundles (Vsb) caused by the alkaline treatment effect.

Fig. 12c shows the image corresponding to the PPF-t/ NaOH5%. The image shows the detachment of the support Fr (Spf) due to the concentration of NaOH in the alkaline treatment. In the same way, the contraction of tissues caused by alkaline treatment can be observed.

Fig. 12d shows the PPF-t/ NaOH6%. The figure shows the detachment of fibrils (Fdt) caused by the deterioration of the surface due to the removal of the lignin and hemicellulose. Polymers that function as coupling agents for the fibrils that constitute the macrofiber. This phenomenon was similar to that described by the authors Cadena et al. [87] who when applying an alkaline treatment with 10 % NaOH observed that increasing the concentration of NaOH in the alkaline treatment increases the breakdown of cellulose and hemicellulose bonds causing the disintegration of fibrils.

## 5 Conclusion

In the research carried out, the effect of alkaline treatment on PPF was studied. The study described the phases of the research that led to traceability from PPF procurement to alkaline treatment implementation and execution. The results of each of the tests performed led to the following conclusions:

❖ The alkaline treatment applied to the PPF generated a Lw that ranged between 16 % and 21 %. However, the difference in Lw between PPF-t/ NaOH5% and PPF-t/ NaOH6% did not exceed 1 %. This feature allows the calculation of the weight of PPF-Ut to be entered into the alkaline treatment in order to have a clear calculation of the PPF-t to be recovered.
❖ The lignin, cellulose, and hemicellulose content of the PPF studied allowed the comparison of the effect of these polymers on the mechanical strength of PPF-Ut and PPF-t. It was concluded that the lignin content of the PPF-t allowed the increase of % Elg. Similarly, the loss of hemicellulose and lignin contributes to the deterioration of the fibrils causing their detachment.
❖ The concentration of NaOH in the alkaline treatment and the exposure time of the PPF in the alkaline solution must be controlled to avoid the detachment of the set of fibrils that make up the macrofiber.
❖ The control of lignin loss allows the interlacing of the fibrils with a positive effect on the mechanical strength of PPF-t. This phenomenon is due to the interaction of lignin free zones that favor their approach and bonding.
❖ Hemicellulose acts as a coupling agent in PPF. Excessive loss of this essential polymer considerably decreases the structural behavior of PPF under external stresses.
❖ SEM observations allowed to identify the effect of alkaline treatment on the surface of the PPF-t. Si bodies exposed by alkaline treatment were visualized. This phenomenon is due to the removal of impurities and Wx present in the surface topography of the PPF. Characteristic evidencing the cleaning carried out by NaOH.
❖ With EDS mapping, the elemental chemistry of PPF can be identified. The presence of O and C in the PPF-t can be determined. Chemical elements that make up cellulose Fr. This phenomenon was due to the visualization of the cellulose fibrils that make up the macrofiber.
❖ SEM visualization of the cross-section of the PPF allowed to identify the influence of alkaline treatment on the conductive tissue. Contractions were observed in the Vsb made up of the Xym and Phm. Contractions caused by the rupture of the Spf present in the Vsb.
❖ SEM analysis of the PPF-t/ NaOH6% cross-section allowed the identification of the Fdt. Effect caused by the removal of lignin, hemicellulose, and Wx due to the high NaOH content in solution.
❖ Alkaline treatment positively influences the mechanical behavior of PPF. However, NaOH concentration and PPF exposure time must be controlled in order to avoid fibril separation due to excessive lignin, and hemicellulose removal.

## CRediT authorship contribution statement

**Oswaldo Hurtado-Figueroa:** Writing – original draft, Validation, Resources, Methodology, Investigation, Formal analysis, Data curation, Conceptualization. **Humberto Varum:** Writing – review & editing, Supervision, Methodology, Formal analysis. **María Isabel Prieto:** Writing – review & editing, Supervision, Data curation. **Romel J. Gallardo Amaya:** Writing – review & editing, Supervision, Data curation. **Alfonso Cobo Escamilla:** Writing – review & editing, Supervision, Methodology, Formal analysis.

## Data availability

Some or all data generated or used during the study are available from the corresponding author by request.

## Funding

This research received no external funding.

## Declaration of Competing Interest

The authors declare that they have no known competing financial interests or personal relationships that could have appeared to influence the work reported in this paper.
